# Autoimmune Cytopenias in Chronic Lymphocytic Leukemia

**DOI:** 10.1155/2013/730131

**Published:** 2013-04-16

**Authors:** Giovanni D'Arena, Roberto Guariglia, Francesco La Rocca, Stefania Trino, Valentina Condelli, Laura De Martino, Vincenzo De Feo, Pellegrino Musto

**Affiliations:** ^1^Onco-Hematology Department, IRCCS Centro di Riferimento Oncologico della Basilicata, 85028 Rionero in Vulture, Italy; ^2^Laboratory of Pre-clinical and Translational Research, IRCCS Centro di Riferimento Oncologico della Basilicata, 85028 Rionero in Vulture, Italy; ^3^Department of Pharmaceutical and Biomedical Sciences, University of Salerno, 84131 Salerno, Italy

## Abstract

The clinical course of chronic lymphocytic leukemia (CLL) may be complicated at any time by autoimmune phenomena.The most common ones are hematologic disorders, such as autoimmune hemolytic anemia (AIHA) and immune thrombocytopenia (ITP). Pure red cell aplasia (PRCA) and autoimmune agranulocytosis (AG) are, indeed, more rarely seen. However, they are probably underestimated due to the possible misleading presence of cytopenias secondary to leukemic bone marrow involvement or to chemotherapy cytotoxicity. The source of autoantibodies is still uncertain, despite the most convincing data are in favor of the involvement of resting normal B-cells. In general, excluding the specific treatment of underlying CLL, the managementof these complications is not different from that of idiopathic autoimmune cytopenias or of those associated to other causes. Among different therapeutic approaches, monoclonal antibody rituximab, given alone or in combination, has shown to be very effective.

## 1. Introduction

Autoimmune phenomena may complicate the clinical course of chronic lymphocytic leukemia (CLL). Autoimmune cytopenias (AC) are much more common than other non hematologic complications, sometimes representing the first appearance of the disease [1–4]. The most frequent autoimmune disorder is hemolytic anemia (AIHA); immune thrombocytopenia (ITP), pure red cell aplasia (PRCA) and autoimmune agranulocytosis (AG) are more rarely seen and probably underestimated, being frequently considered due to disease infiltration of bone marrow or as a consequence of hematologic toxicity of chemotherapy [5–10]. Other autoimmune diseases may be observed in CLL patients, such as bullous pemphigoid, allergic vasculitis, rheumatoid arthritis, systemic lupus erythematosus, ulcerative colitis [2–11].

The incidence of hemic autoimmune complications in CLL is quite different in studies published so far. This is probably due to the fact that it is sometimes difficult to understand the cause of cytopenias in these patients. Overall, the percentage of patients experiencing cytopenia during the course of the disease is estimated from 4.3% to 9.7% [9, 10].  Table 1 summarizes the incidence of the hematological autoimmune disorders in CLL patients.

Among others, a large series of patients was reported by Duek et al. [11]. They analyzed 964 patients from the Israel CLL Study Group, followed for 35 years and found 115 cases showing a single or a combination of autoimmune disorders. Among them, 11 (1.1%) had AIHA at diagnosis, 35 (5–7%) had a direct antiglobulin test (DAT)-positivity without clinical and laboratory evidence of AIHA at diagnosis, 43 (3.7%) developed AIHA during the follow-up (6 following fludarabine therapy), 9 had ITP, two of whom being DAT-positive also and classified as having Evan's syndrome.

More recently, Moreno et al. analyzed 960 Spanish CLL patients followed for 28 years, showing that 70 (7%) patients had AC: 59 (6%) had AIHA, 20 (2%) had ITP, and 1 patient (0.1%) had Evan's syndrome [7]. Zent and Kay analyzed the largest series (1,750 patients with CLL) followed for 10 years at Mayo Clinic. They showed that 75 patients (4.3%) had cytopenias: 2.3% of them had AIHA, 2% ITP and 0.5% PRCA [8]. Finally, focusing only on ITP, Visco et al. found 69 (5%) of cases in a cohort of 1,270 patients retrospectively evaluated [12].

## 2. Diagnostic Criteria of CLL-Associated AC and Prognostic Relevance

Anemia and thrombocytopenia, irrespective of autoimmunity or bone marrow infiltration as cause of cytopenia, define advanced Rai and Binet clinical stage IV and C, respectively [13, 14]. Furthermore, according to the International Workshop on Chronic Lymphocytic Leukemia (IWCLL) guidelines, both AIHA or ITP “poorly responsive to corticosteroids or other standard therapy” are considered as criteria to start treatment for CLL [15]. More recently, Strati and Caligaris-Cappio better addressed the matter of the occurrence of autoimmune disorders as an indicator of therapy in CLL [16]. These authors concluded that simple-refractory and complex autoimmunity have to be considered an indication to treatment of CLL at present.

In light of this, the cause of cytopenia need to be carefully evaluated, despite the lack of standardized clinical and laboratory diagnostic criteria of AC in CLL patients. In the majority of cases, diagnosis is established according to criteria commonly used to diagnose AIHA, ITP, PRCA, and AG in non-CLL patients. However, bone marrow lymphocyte infiltration, splenomegaly or chemotherapy concurrently given may obstacle a correct diagnosis.

Table 2 summarizes the commonly used criteria to diagnose AC in CLL patients, as derived from published papers focusing on this issue.

Controversial results have been reported on the prognostic significance of AC in CLL. In fact, the presence of AIHA was shown to be a poor prognostic indicator in some studies [17, 18]. Among others, Dearden et al. reported that patients without AIHA had a significantly better overall survival and progression free survival with respect to patients with AIHA [5]. On the contrary, Mauro et al, found that AIHA has no effect on survival [19]. Likewise, Kyasa et al. showed that AC does not predict poor prognosis in CLL/small lymphocytic lymphoma (SLL) patients [20]. Visco et al. reported that patients with early onset of AIHA (within 48 months from diagnosis) have a shorter survival and that AIHA is associated with unmutated IgVH status in CLL patients [21]. The same authors analyzed a large series of CLL patients with ITP and showed that this autoimmune complication is associated with unmutated IgVH status and poorer survival [12]. More recently, Moreno et al. reported that CLL patients with advanced clinical stage (Binet C) due to immune mechanism had significantly better survival than patients with cytopenia due to bone marrow infiltration [7]. Moreover, a clear association between AC and other poor prognostic variables in CLL (high leucocyte count, rapid blood lymphocyte doubling time, *β*2-microglobulin and CD38/ZAP-70 expression) was also found.

## 3. Pathogenesis

CLL appears as a malignant disease and a complex immunologic disorder as well [1, 22–24].The cellular origin of CLL remains still unknown. Chiorazzi and Ferrarini suggested that CLL may derive from marginal zone B-cell clonal expansion [25]. Human B-1 cells have been recently also identified and proposed to originate CLL [26, 27]. This small subset of B-cells constitutively produces antibodies and has been connected to autoimmune disorders. Immature B-cells leaving the bone marrow to reach the splenic microenvironment and completing their maturation process need to pass through transitional stages, and, therefore, they are called transitional B cells [28]. All these cells can be positively selected by autoantigen reactivity. However, only a minority of them will successfully complete this process due to crucial checkpoints for controlling autoreactivity.

T-cell function in CLL patients is impaired and, at the same time, B-cells produce an insufficient amount of antibodies inducing hypo-gammaglobulinemia [5, 29–40]. These dysfunctions are a hallmark of CLL since the onset of the disease.  Table 3 summarizes some of the immunologic alterations described in CLL patients.

Infections and a higher rate of second tumors with respect to normal subjects are considered a possible consequence of such a profound immunosuppression. Moreover, T-cell impairment and abnormal interactions between T and B-cells cause a deficiency of the immune surveillance system facilitating the emergence of resting normal B-cell clones, able to produce autoantibodies against erythrocytes, granulocytes and platelets [1–3].

More recently, regulatory T-cells (Tregs) have been evaluated in CLL [41–44]. They were found abnormally expressed in CLL patients and have been considered to be also involved in the escape of autoreactive clones [43, 44]. CLL cells probably deliver inhibitory signals to Tregs preventing the elimination of autoreactive T and B-cells.

The source of autoantibodies involved in autoimmune disorders associated with CLL is still an unresolved question. Some investigators demonstrated that autoantibodies are due to the neoplastic B-cells, while others have suggested that they are produced by resting normal B-cells as a consequence of T-cell disturbance [45–48].

**Figure 1:**
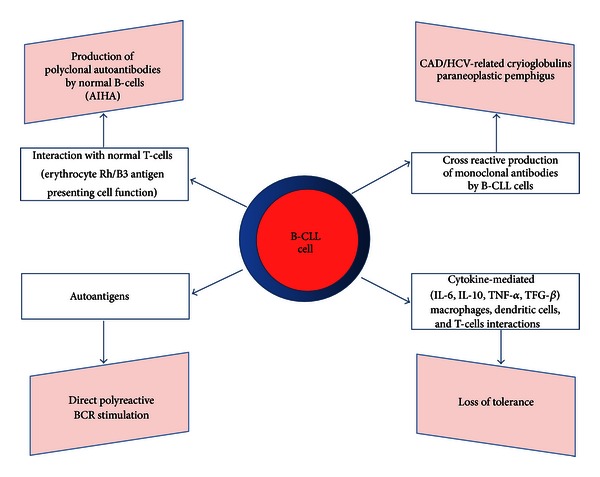
Four main hypotheses of the CLL-associated autoimmune disorders pathogenesis. (1) CLL B-cells may act as either antigen presenting cells and processing cells of red blood cells (RBCs), thus inducing a T-cell response and, in turn, the activation of resting B-cells with the production of polyclonal antibodies against erythrocytes and, ultimately, hemolysis. (2) CLL B-cells may more rarely act as effector cells secreting pathological monoclonal autoantibodies. This is thought to happen in cold agglutinin disease (CAD), hepatitis C virus (HCV)-related cryoglobulins, and paraneoplastic pemphigus. (3) Autoantigens may stimulate B-CLL cells by means of their polyreactive B cell receptor (BCR). (4) Inhibitory cytokines, such as interleukin (IL)-6, IL-10, tumor necrosis factor (TNF), and tumor growth factor (TGF)-*β*, may be produced by B-CLL cells resulting in the loss of tolerance.

Overall, four main mechanisms of autoimmune disease in CLL have been proposed (Figure 1) and recently reviewed by Hodgson et al. [9]. Firstly, a role of efficient antigen presenting cells for leukemic B-cells has been demonstrated in CLL-associated AIHA by Hall et al. [54]. In fact, they showed that CLL cells may act as efficient antigen presenting cells (APCs) inducing a T-cell response that, in turn, induces the subsequent activation of resting normal B-cells and the production of polyclonal autoantibodies. CLL cells may also act secreting inhibitory cytokines that alter immune tolerance, thus facilitating the escape of self-reactive clones. Though rarely, CLL cells may act as effector cells secreting a pathological monoclonal autoantibody. Finally, CLL cells may be stimulated through their polyreactive B-cell receptor (BCR) that recognizes auto-antigens.

Production of monoclonal or polyclonal autoantibodies by B-cells against eryhtroid cells (that may be involved at any stage of differentiation) and, though more rarely, autoantibodies against erythropoietin receptor or the red-cell signaling pathway, are thought to play a key role in the pathogenesis of CLL associated-PRCA [55, 56]. However, an alternative notion supporting the main role of T and NK-cells in suppression of erythropoiesis has also been proposed (reviewed by D'Arena and Cascavilla [6]). In fact, experimental studies showed that the inhibition of erythropoiesis is due to the dysfunction of the so-called *γδ*T cells carrying the Fc receptor for IgG (CD16) [57–64]. These cells were shown to be increased in bone marrow of patients with CLL-associated PRCA and inhibit erythroid growth in vitro [56]. In addition, these cells are not able to support the growth of burst-forming unit-erythrocyte (BFU-E) as usually happens in normal individuals [58–61].

HLA class I proteins inhibit killer-cell inhibitory receptors (KIRs) normally expressed on *γδ*T cells and like NK-cells. Handgretinger et al., analyzing a case of LGL leukemia-associated PRCA, proposed an intriguing model of inhibition of erythropoiesis in PRCA [65]. KIRs and HLA class I antigen ligation inhibits lysis of target cells by NK or *γδ*T cells. These latter lyse cells in a MHC unrestricted manner, thus targeting cells that do not display HLA class I proteins. Usually, BFU-E express HLA class I antigens and, as the maturation of erythroid cells progress, a down-regulation of such antigens is seen. In this way, a clonal expansion of NK-cells or *γδ*T cells is able to induce pro-erythroblast lysis, determining the clinical picture of PRCA.

## 4. Fludarabine-Related Autoimmune AIHA

Fludarabine (FAMP) is the most effective and extensively studied purine analog in B-cell chronic lymphoid malignancies. The combination of FAMP, cyclophosphamide and anti-CD20 monoclonal antibody rituximab (FCR regimen) has emerged as the current standard of care in the treatment of younger CLL patients [66]. AIHA may complicate FAMP therapy [6].  Table 4 summarizes data on the incidence of AIHA in FAMP-treated CLL patients from some of the largest studies published so far. As shown, Di Raimondo et al. reported 13 (11%) cases of AIHA in a cohort of 112 CLL patients treated with FAMP alone [49]. Twelve (21%) out of 59 CLL patients were reported to develop AIHA after FAMP treatment by Myint et al. [50]. In addition, 6 out of 8 patients re-treated with FAMP after hemolysis control developed an exacerbation of their AIHA. In a series of 1,203 CLL patients studied at a single institution for more than 10 years, Mauro et al. found 3 cases of AIHA (2.5%) in the group of patients treated with FAMP and prednisone versus 10 cases (1.8%) observed in the group of patients who had received chlorambucil and prednisone [19]. Thirty-eighth cases of AIHA occurred in a French multicenter randomized trial comparing FAMP alone versus cyclophosphamide, doxorubicin and prednisone (CAP) or cyclophosphamide, doxorubicin, vincristine and prednisone (CHOP), as first-line treatment of advanced CLL patients; no statistically significant different distribution in the three arms was observed [67]. Catovsky and Richards reviewed data from MRC CLL trials in the last 20 years [51]. The incidence of AIHA was 8.6% in untreated patients, while it was 11% in treated patients, the majority of them receiving alkylating agents. The same group reported the final results of the LFR CLL4 multicenter trial in which patients with A progressive, B and C Binet stage CLL were randomized to receive chlorambucil, FAMP alone or FAMP plus cyclophosphamide (FC) [68]. AIHA was less common in the FC group than in other groups, thus suggesting a protective role of cyclophosphamide when combined with FAMP. The German CLL study group also reported a lower incidence of AIHA with FC compared to FAMP alone [69].

Concerning the use of rituximab, Borthakur et al. performed a retrospective analysis of 300 patients treated at MD Anderson Cancer Center with FCR [52]. They found that 19 (6.5%) of them developed AC on or after therapy: 17 (5.8%) patients experienced AIHA and 2 (0.7%) PRCA. Of interest, AIHA occurred despite DAT negativity in 60% of cases, as FCR may mask DAT positivity below the threshold of detection of commonly used tests. 

Finally, Hallek et al. reported data from 408 CLL patients enrolled in a randomized trial comparing FCR to FC; no difference (1% versus <1%) was found in terms of AIHA incidence [53].

Taken together, these data allow to conclude that: (1) AIHA is part of the natural history of CLL that presents by itself a risk for autoimmune hemolysis; (2) early reports of excess of AIHA in CLL patients treated with FAMP were in the context of advanced and heavily pretreated disease; (3) recent randomized trials in previously untreated CLL patients showed that FAMP is no more hemolytic than other agents and that this complication is limited by adjunction of cyclophosphamide, thus suggesting that this purine analog can be safely used for patients with AC complicating CLL and who required chemotherapy. Rituximab could also play a role in this setting.

## 5. Treatment of CLL-Associated AC

Due to the relatively small number of CLL patients with AC, no prospective trial has investigated the specific treatment of these complications. Indeed, available data only derive from retrospective series and case reports. In general, AC, when present, should be treated before deciding whether therapy for CLL is needed and CLL specific treatment should be applied only when standard immunosuppressive therapy has failed [15, 70]. 

The management of these disorders in CLL patients is not different from that of idiopathic forms or those associated with other causes of AC [71–73]. Prednisone is usually given as first-line therapy at the standard dose of 1 mg/kg body weight daily for 4 to 12 weeks, followed by a gradual doses tapering. Higher doses of corticosteroids have been also given. Splenectomy, that still remains a standard second-line treatment for adults with idiopathic AIHA and ITP [72, 73], should be considered in CLL-associated AIHA and ITP with more caution. Furthermore, splenectomy is more and more challenged by other treatments, such as thrombopietin (TPO)-mimetics romiplostim and eltrombopag, whose possible efficacy in CLL-associated ITP has been recently reported and is currently under further investigation [74, 75]. Vincristine, cyclophosphamide, cyclosporin-A and intravenous immunoglobulis have been also used in small series and case reports and may be useful in particular circumstances.

Monoclonal antibodies rituximab (directed against CD20 antigen) and alemtuzumab (directed against CD52 antigen) have more recently emerged as potential curative treatment of CLL-associated AC [76]. D'Arena et al. reviewed data on the use of such antibodies to treat CLL-associated AIHA and reported a personal experience on the successful use of rituximab in small series of ITP and PRCA patients with CLL [4, 6, 77, 78]. Positive results with alemtuzumab for AIHA in CLL patients have been reported by Karlsson et al. in five patients resistant to other conventional treatments [79]. Of note, the best results in CLL-associated AIHA were probably obtained with the RCD (Rituximab, Cyclophosphamide, and Dexamethasone) regimen described by Gupta et al. (100% response in 8 patients) [80], results recently confirmed on a larger series of 48 CLL patients with AC by Rossignol et al., who achieved an overall response close to 90%, followed, however, by about 40% of relapses [81]. 

Concerning AG, some patients may benefit form a G-CSF treatment. [70].

## 6. Conclusions

Cytopenias are frequently observed in CLL as a consequence of bone marrow infiltration or myelosuppression due to chemotherapy. However, despite less commonly, AC may complicate the clinical course of CLL at any time and, sometimes, may anticipate its diagnosis. Of interest, the use of chemoimmunotherapy probably reduce the incidence of AIHA in CLL, but it should be taken into account that DAT negativity, in these patients, does not exclude the presence of this complication. While AIHA is relatively easy to diagnose, CLL-associated ITP, PRCA, and AG require more attention, as concomitant lymphocyte infiltration may cause a diagnostic mistake. For that reason, ITP, PRCA, and AG in patients with CLL are probably underestimated.

It is very important to diagnose AC because they require immunosuppressive therapy. In the presence of AC, CLL specific treatment should be applied only when such an approach has failed. Conventional immunosuppressive therapy is usually used to treat these disorders. Rituximab alone or combined with other immunosuppressive agents, currently appears to be the preferable therapeutic option.

## Figures and Tables

**Table 1:** Reported incidence of autoimmune cytopenia complicating CLL.

Autoimmune cytopenia	Incidence
AIHA* [1–5, 7–10]	4.5–11%
ITP [1, 2, 10, 12]	2–5%
PRCA [1, 2, 6, 10]	<1%
AG [1, 2, 7, 10]	<1%

AIHA: autoimmune hemolytic anemia.

ITP: immune thrombocytopenia.

PRCA: pure red cell aplasia.

AG: autoimmune granulocytopenia.

*A positive direct antiglobulin test (DAT) without clinically evidence of hemolysis may be found in 7–14% of patients [5].

**Table 2:** Recommendations for the diagnosis of CLL-associated autoimmune cytopenias.

Autoimmune Hemolytic Anemia	
(1) Positive DAT*	
(2) Reticulocytosis	
(3) Elevated serum LDH	
(4) Elevated serum indirect bilirubin*	
(5) Reduce serum haptoglobin*	
(6) Erythroid hyperplasia in bone marrow	

Autoimmune Pure Red Cell Aplasia	
(7) Severe normocromic-normocytic anemia*	
(8) Reticulocytopenia*	
(9) Erythroid precursors ≤1% of bone marrow cells*	
(10) No parvovirus B19 infection by polymerase chain reaction assay*	
(11) DAT negativity*	
(12) No presence of hemolysis (normal haptoglobin, unconjugated bilirubin, LDH)*	
(13) More than 4–8 weeks from the last chemotherapy infusion*	

Immune Thrombocytopenia	
(1) Rapid and “unexplained” fall in the platelet count*	
(2) Augmented number of megakaryocytes in the bone marrow*	
(3) More than 4–8 weeks from the last chemotherapy infusion*	

Autoimmune granulocytopenia	
(1) Persistent and “unexplained” neutropenia*	
(2) Decreased or absent granulocyte precursors in bone marrow*	
(3) Presence of anti-neutrophilantibodies	
(4) More than 4–8 weeks from the last chemotherapy infusion*	

DAT: direct antiglobulin test.

LDH: lactatedeydrogenase serum levels.

Note that some of these criteria can be not always applicable for patients with CLL, in particular in the case of AIHA (i.e., absence of recticulocytosis due to bone marrow infiltration and/or elevated LDH without AIHA in case of aggressive CLL; ITP is not always rapid). Furthermore, DAT may be negative in patients with AIHA complicating-CLL.

*Marks criteria that, in our opinion, should be considered more relevant for AC diagnosis in patients with CLL.

**Table 3:** Immune defects in CLL.

T-cell
Increased circulating number [29, 31–33, 36, 39, 40]
Decresed CD4/CD8 ratio [29, 36]
Th2 polarization [29, 35, 36]
Impaired immunological synapse [38]
Increased circulating number of Tregs [39, 41–44]
B-cell
Hypogammaglobulinemia [33, 39, 40]
Poor response to vaccination [31, 37]
NK cell
Increased circulating number [39]
Reduced killing activity [30, 39]
Lack of granules [30]
Neutrophils
Reduced phagocytic and bactericidal function [31, 32, 39]
Abnormal migration and chemotaxis [39]

**Table 4:** Reported incidence of AIHA in fludarabine containing regimen treated patients with CLL.

Reference	No. of AIHA cases/no. of patients evaluated	Relative number of AIHA (%)	Type of therapy given
Di Raimondo et al. [49]	13/112	11	FAMP alone
Myint et al. [50]	12/59	21	FAMP alone
Mauro et al. [19]	3/121 10/559	2.5 1.8	FAMP + prednisone Chlorambucil + prednisone
Catovsky and Richards [51]	47/387 21/194 9/198	12 11 5	Chlorambucil FAMP FC
Borthakur et al. [52]*	All cases 17/300 DAT-positive 3/300	All cases 5.8 DAT-positive 1.4	FCR
Hallek et al. [53]	4/249 3/404	1% <1%	FC FCR

*Three out 17 patients with AIHA had a positive-DAT, while the remaining 14 patients had a negative-test.
